# Health care costs of injury in the older population: a prospective multicentre cohort study in the Netherlands

**DOI:** 10.1186/s12877-020-01825-z

**Published:** 2020-10-21

**Authors:** Marjolein van der Vlegel, Juanita A. Haagsma, A. J. L. M. Geraerds, Leonie de Munter, Mariska A. C. de Jongh, Suzanne Polinder

**Affiliations:** 1 Department of Public Health, Erasmus MC, University Medical Center Rotterdam, Rotterdam, The Netherlands; 2 Department Trauma TopCare, ETZ Hospital, Tilburg, The Netherlands

**Keywords:** Health care costs, Wounds and injuries, Aged, Critical care, Generalized linear model

## Abstract

**Background:**

With the ageing population, the number of older trauma patients has increased. The aim of this study was to assess non-surgical health care costs of older trauma patients and to identify which characteristics of older trauma patients were associated with high health care costs.

**Methods:**

Trauma patients aged ≥65 years who were admitted to a hospital in Noord-Brabant, the Netherlands, were included in the Brabant Injury Outcome Surveillance (BIOS) study. Non-surgical in-hospital and up to 24- months post-hospital health care use were obtained from hospital registration data and collected with the iMTA Medical Consumption Questionnaire which patients completed 1 week and 1, 3, 6, 12 and 24 months after injury. Log-linked gamma generalized linear models were used to identify cost-driving factors.

**Results:**

A total of 1910 patients were included in the study. Mean total health care costs per patient were €12,190 ranging from €8390 for 65–69 year-olds to €15,550 for those older than 90 years. Main cost drivers were the post-hospital costs due to home care and stay at an institution. Falls (72%) and traffic injury (15%) contributed most to the total health care costs, although costs of cause of trauma varied with age and sex. In-hospital costs were especially high in patients with high injury severity, frailty and comorbidities. Age, female sex, injury severity, frailty, having comorbidities and having a hip fracture were independently associated with higher post-hospital health care costs.

**Conclusions:**

In-hospital health care costs were chiefly associated with high injury severity. Several patient and injury characteristics including age, high injury severity, frailty and comorbidity were associated with post-hospital health care costs. Both fall-related injuries and traffic-related injuries are important areas for prevention of injury in the older population.

**Supplementary information:**

**Supplementary information** accompanies this paper at 10.1186/s12877-020-01825-z.

## Background

Globally, the number of older people will increase substantially in the coming decades [1]. The proportion of the population over 65 years old in the Netherlands is expected to increase from 18.8% in 2018 to 26% in 2040 [2]. Moreover, the proportion of those aged 80 years and older is expected to double (4.5% in 2018 [3] to 8.7% in 2040 [4]). At present, nearly 50% of all trauma patients in the Netherlands are 65 years or older and more than 25% are older than 80 years [5]. Additionally, an increase in injury in the older population can be expected because of the independence and active lifestyles of older people. Since people as they age become more vulnerable and are more likely to experience comorbidities, they are at greater risk of adverse health outcomes [6, 7].

Compared to the younger trauma population, older patients have longer hospital stays, have higher risk of complications, have higher health care consumption after discharge and are more likely to die due to their injury [5, 7, 8]. The high incidence of comorbidities and frailty in the older population are factors contributing to these outcomes [6, 8]. As a consequence, the need for long-term care and the associated health care costs for this population are increasing, presenting a high economic burden to individuals and society.

Several studies examined the health care costs of adults trauma patients [9–11]. In the older trauma population, fewer costs of all cause trauma studies exist. Previous research on costs of injury in the older population has mainly focused on falls [12–14]. Other studies have focused on costs of specific injury types like hip fractures [15–17] and traumatic brain injury [18]. In general, studies have demonstrated that frailty [19–21] and comorbidity [22] are associated with increased health care utilization and costs. However, studies within the trauma population are scarce. Two studies within the older trauma population found no association between frailty status and hospital costs [23, 24] The majority of costs of injury studies present only intramural health care costs, despite the fact that especially the long-term extramural health care costs can be high for the older injury population.

In this study we provided a detailed overview of non-surgical health care costs of the older trauma population in the Netherlands for the whole spectrum of injuries. We aimed to assess short-term, in-hospital and long-term, post-hospital health care costs and to identify determinants of health care consumption and costs of trauma patients aged ≥65 years.

## Methods

### Study design and population

This study is part of the Brabant Injury Outcome Surveillance (BIOS) study, a prospective longitudinal cohort study. The design of the BIOS study has been described in detail in the published research protocol [25]. Briefly, the BIOS was conducted in ten hospitals of the Dutch Noord-Brabant region. Injured patients (≥65 years) who were admitted to a ward or Intensive Care Unit (ICU) through the Emergency Department (ED) between August 2015 and November 2016 were eligible for inclusion in this study. Patients were excluded if they had pathological fractures, had insufficient knowledge of the Dutch language or had no permanent address. If at least one questionnaire was completed and if hospital registry data on in-hospital care were available, patients were included in this study. If a patient was not able to fill in the questionnaire, a proxy informant (e.g. a family member) could fill in the questionnaire. The BIOS study has been approved by the Medical Ethics Committee Brabant (NL50258.028.14) and all participants and proxy informants signed informed consent for participation.

### Patient and injury characteristics

During a follow-up of 24 months, data were collected with repeated questionnaires at 1 week and 1, 3, 6, 12, and 24 months after injury. The first questionnaire included items regarding socio-demographics (e.g. age and sex) and included items regarding the presence of pre-existing conditions and frailty. To assess comorbidity, the questionnaire included items on 14 conditions: heart disease, vascular disease, lung disease, consequences of a stroke, neurological disease, kidney disease, diabetes mellitus, osteoporosis, dementia, psychiatric disorder (depression, anxiety disorder), herniated disk or other severe back problems, arthritis, rheumatism and cancer. The pre-injury frailty status of a patient was assessed with the 15-item Groningen Frailty Index (GFI). A score ≥ 4 on a scale of 0–15 was considered to indicate frailty [26].

Injury related characteristics, including the Abbreviated Injury Scale (AIS-90, update 2008) [27] and the Injury Severity Score (ISS) [28] were registered in the Brabant Trauma Registry (BTR). The AIS provides a severity code for each body region. The overall trauma severity was assessed by the ISS, which is a score ranging from 1 to 75. The ISS is calculated by squaring and summing the highest AIS severity codes in each of the three most severely injured body regions.

The Dutch CTG or CBV classification system were used for the registration of surgical interventions in hospitals. Hospital registration systems were linked to our dataset to identify patients with surgical interventions. This included all types of surgical interventions, varying from smaller interventions like a wound excision to larger interventions like hip replacement surgery.

### Health care consumption and cost calculation

Detailed information on the collection of health care use data and the health care cost calculations are described in a previous study [9]. Health care use data were collected from hospital registries and the iMTA Medical Consumption Questionnaire (iMCQ). The iMCQ was included in the questionnaires at 1,3, 6, 12 and 24 months after injury and included items related to intramural (e.g. stay at a hospital) and extramural (e.g. day treatment at an institution) health care use, related to the trauma.

Unit costs of health care services were retrieved from a cost-reference manual, presented in Table A.1 [29], except for unit costs of diagnostics, which were retrieved from hospital price lists, previous research and the Dutch Healthcare Authority (NZa) [30–37]. Health care costs were calculated by multiplying health care use with cost per unit. If data for a service was missing, health care utilization was set to 0 for the calculation of total in-hospital costs, total post-hospital costs and total health care cost. Costs were inflation-adjusted to 2017 euro using consumer price index rates.

The medical costs were divided into in-hospital and post-hospital costs. In-hospital costs were transportation to the ED, stay at a hospital ward, stay at ICU and diagnostic procedures. Post-hospital costs were stay at an institution (nursing home, rehabilitation centre or psychiatric institution), day treatment at an institution, home care (domestic care, help with all day activities or nursing) and contact with practitioners (general practitioner, company doctor, psychologist, social worker, physiotherapist, occupational therapist, speech therapist or dietician physiotherapist). Other hospital costs, such as surgery costs, were not included.

### Statistical data analysis

Chi-square test of homogeneity (categorical) and Mann-Whitney *U* test (continuous) were conducted to test for differences between participants and non-participants regarding demographic and injury-related characteristics. Descriptive statistics (mean and standard deviation (SD)) were used to determine the costs of transportation to the ED, stay at a hospital ward, stay at ICU, diagnostic procedures and stay at an institution, day treatment at an institution, home care and contact with practitioners. The total in-hospital costs, total post-hospital costs and total health care costs were determined for subgroups for age, sex, cause of trauma, type of injury, ISS, number of comorbidities and frailty status.

We analysed various determinants of health care costs in generalized linear models (GLM) with gamma distribution and log link function. This is a commonly used method for cost data since costs are constrained to be positive and the distribution is right skewed [38]. To account for uncertainty associated with missing data in regression analysis, we used multiple imputation by chained equations (MICE) to impute missing values of comorbidities and frailty [39]. Comorbidities were unknown for 33 participants (1.7%). The GFI questionnaire including 15 items, was partially completed by 215 (11.3%) participants and not completed by 296 (15.5%) participants. We applied multiple imputation to the missing item scores as advised by Eekhout et al. (2014) if the questionnaire was partially completed [40]. In the analyzed sample, 149 (7.8%) participants had 1 missing GFI value, 27 (1.4%) participants had 2 missing GFI values, 19 (1.0%) participants had 3–5 missing GFI values and 20 (1.0%) participants had more than 5 missing GFI values. If the questionnaire was not completed, the participants (*n* = 296, 15.5%) were placed in an ‘unknown frailty status’ category. The following variables were included in the imputation model to impute the missing data for comorbidity and GFI values: sex, age, living situation (at home or somewhere else), cause of trauma, use of proxy respondent, ISS, type of injury (e.g. hip fracture, traumatic brain injury, pelvic injury), number of comorbidities and available GFI values. The dataset was imputed 30 times with 10 iterations. One patient had an unknown ISS and was excluded from the analysis. In the regression analysis, age was categorized into 5-year age groups and ISS was categorized as 1–3, 4–8, 9–15 and ≥ 16. Cause of trauma was categorized as: home and leisure, traffic, sport and other (e.g. occupational, self-harm, interpersonal violence, unknown), frailty status as not frail (GFI score 0–3), frail (GFI score 4–15) or unknown frailty status and number of comorbidities as having no comorbidity, one comorbidity, two comorbidities or three or more comorbidities. All statistical analyses were performed in SPSS version 24.0, except for the multiple imputation which was performed in R version 3.6.0, with the R package MICE [41]. A *p*-value < 0.05 was considered statistically significant.

## Results

### Study population

In total, 1910 (34.7%) trauma patients were included in this study (see additional file 2). Table 1 shows the characteristics of participants and non-participants. The mean age of participants was 78.3 (SD 8.4) years and the majority of the patients was female (*n* = 1165, 61.0%). The most common cause of trauma were falls (*n* = 1239, 64.9%) and the most common type of injury were hip fractures (*n* = 792, 41.5%). Of all participants with a completed GFI questionnaire, 34.7% (*n* = 662) were considered to be frail and 80.1% (*n* = 1496) had one or more comorbidities. Participants were significantly younger (78.3 years vs 81.2 years respectively), more often male (39% vs 32.6%) and had a higher ISS (7.0 (SD 3.9) vs 6.6 (SD 4.1)), compared to non-participants. Cause of trauma also differed significantly between participants and non-participants.

**Table 1:** Characteristics of the study population, participants and non-participants

	Participants	Non-participants	P-value
Participants, n	1910	3594	
Sex, n (%)			< 0.001
*Male*	745 (39.0)	1170 (32.6)	
*Female*	1165 (61.0)	2424 (67.4)	
Age, mean (SD)	78.3 (8.4)	81.2 (8.1)	< 0.001
Age group, n (%)			
*65–69*	378 (19.8)	376 (10.5)	
*70–74*	321 (16.8)	451 (12.5)	
*75–79*	353 (18.5)	613 (17.1)	
*80–84*	373 (19.5)	793 (22.1)	
*85–89*	281 (14.7)	804 (22.4)	
* ≥ 90*	204 (10.7)	557 (15.5)	
Cause of trauma, n (%)^a^			< 0.001
*Home and Leisure: Falls*	1239 (64.9)	2330 (64.8)	
* Home and Leisure: Other*	205 (10.7)	217 (6.0)	
*Traffic*	381 (19.9)	374 (10.4)	
*Sport*	41 (2.1)	23 (0.6)	
*Other*^1^	36 (1.9)	52 (1.4)	
Most common types of injury, n (%)^2^			
*Hip fracture*	792 (41.5)	1375 (38.3)	0.020
*TBI*	489 (25.6)	917 (25.5)	0.944
* Pelvic injury*	137 (7.2)	175 (4.9)	< 0.001
*Tibia, complex foot or femur fracture*	169 (8.8)	300 (7.6)	0.526
*Shoulder and upper arm injury*	156 (8.2)	318 (8.8)	0.392
*Rib fracture*	173 (9.1)	222 (6.2)	< 0.001
*Radius, ulna, hand fracture*	98 (5.1)	165 (4.6)	0.371
ISS, mean (SD) ^b^	7.0 (3.9)	6.6 (4.1)	< 0.001
ISS, n (%)^b^			
*< 4*	345 (18.1)	774 (21.5)	
*4–8*	526 (27.5)	843 (23.5)	
*9–15*	979 (51.3)	1606 (44.7)	
*≥ 16*	59 (3.1)	103 (2.9)	
Frailty status, n (%)^c^			
*Not frail (GFI 0–3)*	907 (47.4)	n.a.	
*Frail (GFI ≥4)*	662 (34.7)	n.a.	
*Unknown*	296 (15.5)		
Comorbidity status, n (%)^d^			
*No comorbidity*	381 (19.9)	n.a.	
*One comorbidities*	565 (29.6)	n.a.	
*Two comorbidities*	408 (21.4)	n.a.	
*Three or more comorbidities*	523 (29.4)	n.a.	
Died during study period, n (%)	256 (13.4)	n.a.	

*ISS* Injury Severity Score, *SD* standard deviation, *TBI* traumatic brain injury, *n.a.* not available

^a^ participants: 8 missing values (0.4%); non participants: 598 (16.6%)

^b^ participants: 1 missing values (0.1%); non participants: 268 (7.5%)

^c^ 215 (11.3%) participants completed the GFI questionnaire partially. For 170 (8.9%) of the patients with missing values for some GFI items, it could be determined with certainty whether they had an GFI score of 0–3 or ≥ 4

^d^ participants: 33 missing values (1.7%)

^1^includes occupational injuries, self-harm, interpersonal violence and unspecified injuries

^2^only the most common injury types (number of cases > 5%) are shown

### Overview of in-hospital and post-hospital costs

Table 2 provides an overview of mean in-hospital and post-hospital costs per component. Total in-hospital costs were mainly driven by costs of stay at a ward (€3610) and total post-hospital costs were mainly driven by home care (€4870) and staying at an institution (€2000). Increasing costs with age can be mainly attributed to these increasing post-hospital costs of staying at an institution and home care. Mean costs per person of stay at an institution increased from €740 in the 65–69 years age group to €5370 in the ≥90 years age group. Mean costs per person of homecare also increased with age and were more than five times higher for ≥90 years old (€9680) compared to 65–69 years old (€1750).

**Table 2:** Detailed overview of mean (SD) health care costs by age group in 2017 €

	In-hospital costs				Post-hospital costs			
	Ambulance transport *N* = 1910	ICU N = 1910	Ward *N* = 1903	Diagnostics *N* = 1362	Stay in institution *N* = 1351	Day treatment *N* = 1555	Home care *N* = 1671	Practitioner visit *N* = 1674
Total	620 (400)	170 (720)	3610 (4340)	1090 (1590)	2000 (6360)	310 (1590)	4870 (16160)	950 (1500)
Age								
*65–69*	570 (390)	260 (970)	2860 (4790)	1140 (1570)	740 (3990)	270 (1660)	1750 (7610)	1190 (1680)
*70–74*	580 (400)	240 (840)	3100 (4020)	1200 (1140)	940 (4960)	380 (1900)	2350 (9220)	1160 (1680)
*75–79*	620 (460)	190 (720)	3500 (4200)	1300 (2860)	1530 (5300)	140 (820)	4100 (10360)	980 (1300)
*80–84*	650 (520)	140 (600)	4270 (4710)	1080 (870)	3520 (8040)	430 (1600)	6320 (13550)	9800 (1490)
*85–89*	650 (160)	80 (440)	4200 (4460)	960 (920)	3210 (7370)	500 (2090)	8590 (20860)	630 (1380)
*≥ 90*	640 (180)	80 (420)	3950 (2770)	690 (500)	5370 (10320)	130 (890)	9680 (34610)	440 (940)

All costs are rounded to 10 EUR

### Total costs by cause of injury

**Fig. 1:**
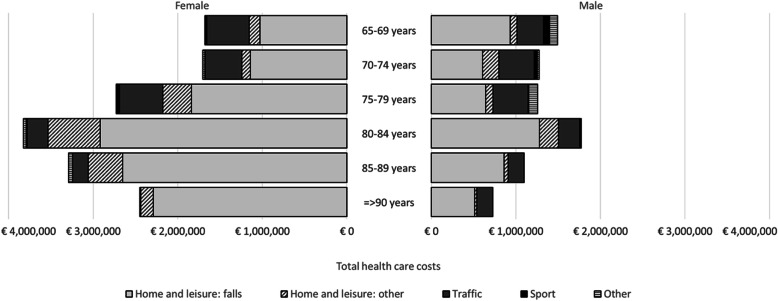
Total health care costs by cause of trauma by age group in 2017 €

Most patients (*n* = 1444, 75.6%) had a cause of injury in the home and leisure category, consisting mainly of falls as a mechanism of injury (n = 1239, 64.9%). Falls contributed to 72% of the total health care costs with mean costs per patient of €13,480 (SD €20,700), but this percentage differed by age category and sex. For female patients aged 65–69, falls contributed to 61% of the total health care costs, whereas for female patients older than 90 falls contributed to 94% of the total health care costs. For males, falls contributed from 48 to 79% to the total health care costs. Patients with traffic related injuries contributed to 15% of the total costs, with mean costs per patient of €9610 (€13,760). Traffic injuries were more prevalent in males than females and contributed to 23% of the total health care costs against 12% for females (Fig. 1).

### Factors associated with health care costs

Overall, mean costs per patient were €12,190 (SD €18,690) (Table 3). Mean in-hospital costs per patient were €5430 (SD €4850) and mean post-hospital health care costs per patient were €7270 (SD €17,760). The total health care costs increased with age from €8390 in the 65–69 years age group to €15,550 in the ≥90 years age group. In-hospital costs were comparable between men and women, while post-hospital costs were higher for women (additional file 4). Women were more likely to sustain a hip fracture, be frail and have comorbidities while men were more likely to be admitted to the ICU (additional file 5).

**Table 3:** Mean health care costs per person by determinant in 2017 €

	In-hospital costs € (SD)	Post-hospital costs € (SD)	Total costs € (SD)
Total	5430 (4850)	7270 (17760)	12,190 (18690)
Age, (years)			
*65–69*	4710 (5610)	3760 (9640)	8390 (12160)
*70–74*	4990 (4670)	4460 (11010)	9280 (12980)
*75–79*	5510 (5110)	6130 (11920)	11,280 (13540)
*80–84*	6150 (4890)	9630 (15780)	15,010 (17070)
*85–89*	5850 (4590)	10,920 (21790)	15,610 (22020)
*≥ 90*	5430 (2880)	12,000 (35960)	15,550 (33740)
Sex			
*Male*	5360 (5610)	5140 (13720)	10,220 (15220)
*Female*	5480 (4300)	8660 (19860)	13,450 (20510)
Cause of trauma			
*Home and leisure: falls*	5710 (5280)	8460 (19860)	13,480 (20700)
*Home and leisure: other*	5350 (3040)	7120 (16180)	11,670 (16120)
*Traffic*	4870 (4230)	4850 (12370)	9610 (13760)
*Sport*	3440 (1370)	1500 (3840)	4940 (4470)
*Other* ^*a*^	4710 (5410)	2770 (4640)	7490 (7400)
Type of injury ^b^			
*Hip fracture*	5940 (3650)	10,540 (23580)	15,490 (23510)
*TBI*	4940 (6170)	3900 (11140)	8670 (12990)
*Pelvic injury*	6460 (6270)	7570 (14000)	13,640 (15270)
*Tibia, complex foot or femur fracture*	6050 (6310)	6910 (13640)	12,390 (17270)
*Shoulder and upper arm injury*	5250 (3750)	6500 (12130)	11,330 (13080)
*Radius, ulna, hand fracture*	5710 (5660)	5500 (10470)	10,540 (12980)
*Rib fracture*	5840 (4820)	3470 (9250)	9250 (11310)
ISS			
*< 4*	3710 (3490)	4020 (10810)	7590 (11580)
*4–8*	4990 (5210)	5630 (11970)	10,280 (14320)
*9–15*	6070 (7200)	9450 (22020)	14,710 (22190)
*≥ 16*	8770 (6780)	6140 (12590)	14,590 (16400)
Comorbidity status			
*No comorbidity*	4250 (2940)	4030 (10000)	8190 (11200)
*One*	5610 (5800)	5280 (11030)	10,670 (13100)
*Two*	5270 (4310)	8520 (20550)	13,190 (20820)
*Three or more*	6220 (5140)	11,020 (24780)	15,810 (24890)
Frailty status			
*Not frail (GFI 0–3)*	4850 (5100)	4800 (10440)	9530 (12670)
*Frail (GFI ≥ 4)*	5850 (4550)	9870 (24770)	14,330 (24270)
*Unknown*	6350 (4770)	10,030 (18690)	15,970 (19490)

All costs are rounded to 10 EUR. Values are based on complete data

*SD* Standard Deviation, *ISS* Injury Severity Score, *TBI* traumatic brain injury

^a^ Includes occupational injuries, self-harm, interpersonal violence and unknown causes of trauma

^b^ Only the 7 most common types of injuries are shown. Some patients have multiple injuries and are therefore in more than one group

Table 4 provides multivariable models for in-hospital costs, post-hospital costs and total costs. The unadjusted models can be found in additional file 3. Older age was independently associated with higher costs, especially for post-hospital costs, where mean health care costs of patients older than 90 years were 2.48 (1.78–3.46) times higher compared to patients aged 65–69. Mean health care costs of traffic-related injuries did not differ significantly from costs of leisure-related injuries. However, mean health care costs of patients with sport-related injuries were significantly lower compared to patients with leisure-related injuries. Both in-hospital and post-hospital costs increased significantly with higher injury severity. Compared to patients with an ISS 1–4, total health care costs were 1.75 (1.50–2.04) and 2.36 (1.84–3.03) times higher for patients with respectively an ISS of 9–15 and ≥ 16. In comparison to patients without comorbidities, mean costs of patients suffering from one, two and three or more comorbidities were respectively 1.14 (1.01–1.29), 1.36 (1.19–1.56) and 1.58 (1.39–1.80) higher. Being frail was also associated with both higher in-hospital costs (1.11 [1.04–1.19]) and post-hospital costs (1.39 [1.14–1.69]) compared to not being frail, although no significant association was found for the total costs. In the multivariable model, being female was slightly associated with lower in-hospital costs (0.94 [0.89–0.99]), while post-hospital costs were significantly higher for women (1.34 [1.14–1.57]).

**Table 4:** Associations with in-hospital, post-hospital and total health care costs based on generalized linear models

	In-hospital^a^		Post hospital^b^		Total^a^	
	Exp[β] (95% CI)	p	Exp[β] (95% CI)	p	Exp[β] (95% CI)	p
Age						
*65–69*	ref		ref		ref	
*70–74*	1.07 (0.98–1.16)	0.16	1.14 (0.89–1.47)	0.30	1.09 (0.95–1.24)	0.24
*75–79*	1.10 (1.01–1.20)	0.04	1.28 (1.00–1.62)	0.05	1.19 (1.05–1.36)	0.01
*80–84*	1.24 (1.13–1.35)	< 0.001	1.97 (1.54–2.51)	< 0.001	1.53 (1.34–1.74)	< 0.001
*85–89*	1.15 (1.04–1.27)	0.004	2.19 (1.66–2.90)	< 0.001	1.51 (1.30–1.75)	< 0.001
*≥ 90*	1.06 (0.95–1.17)	0.33	2.48 (1.78–3.45)	< 0.001	1.41 (1.19–1.65)	< 0.001
Gender						
*Male*	ref		ref		ref	
*Female*	0.94 (0.89–0.99)	0.03	1.34 (1.14–1.57)	< 0.001	1.09 (1.00–1.19)	0.04
Cause of trauma						
*Home and leisure: falls*	ref		ref		ref	
*Home and leisure: other*	0.94 (0.87–1.03)	0.20	0.82 (0.64–1.05)	0.12	0.88 (0.77–1.00)	0.05
*Traffic*	0.94 (0.87–1.01)	0.07	0.96 (0.79–1.18)	0.72	0.95 (0.85–1.06)	0.39
*Sport*	0.71 (0.59–0.86)	< 0.001	0.38 (0.21–0.67)	0.001	0.54 (0.40–0.71)	< 0.001
*Other*	0.87 (0.73–1.04)	0.12	0.49 (0.31–0.80)	0.004	0.72 (0.55–0.95)	0.02
Type of injury						
*No Hip fracture*	ref		ref		ref	
*Hip fracture*	0.86 (0.78–0.94)	0.001	1.40 (1.07–1.83)	0.02	1.09 (0.94–1.25)	0.25
ISS						
*< 4*	ref		ref		ref	
*4–8*	1.38 (1.27–1.49)	< 0.001	1.52 (1.19–1.93)	0.001	1.47 (1.30–1.66)	< 0.001
*9–15*	1.79 (1.62–1.98)	< 0.001	1.58 (1.17–2.14)	0.003	1.75 (1.50–2.04)	< 0.001
*≥ 16*	2.45 (2.08–2.88)	< 0.001	2.34 (1.48–3.70)	< 0.001	2.36 (1.84–3.03)	< 0.001
Frailty status						
*Not frail (GFI 0–3)*	ref		ref		ref	
*Frail (GFI ≥ 4)*	1.11 (1.04–1.18)	0.003	1.38 (1.13–1.67)	0.001	1.09 (0.99–1.21)	0.09
*Unknown*	1.21 (1.12–1.31)	< 0.001	1.61 (1.29–2.01)	< 0.001	1.36 (1.21–1.54)	< 0.001
Comorbidity status						
*No comorbidities*	ref		ref		ref	
*1 comorbidity*	1.21 (1.12–1.31)	< 0.001	1.09 (0.87–1.37)	0.44	1.14 (1.01–1.29)	0.04
*2 comorbidities*	1.15 (1.06–1.25)	0.001	1.58 (1.24–2.03)	< 0.001	1.36 (1.19–1.56)	< 0.001
*≥ 3 comorbidities*	1.32 (1.22–1.43)	< 0.001	2.01 (1.57–2.57)	< 0.001	1.58 (1.39–1.80)	< 0.001

Adjusted for all variables in the table

*CI* confidence interval, *ISS* Injury Severity Score, *GFI* Groningen Frailty Index

^a^ Based on 1909 cases

^b^ Based on 1369 cases

Patients with a hip fracture were significantly older, more often female, had a higher ISS, were more often frail and with comorbidity and had a longer length of hospital stay compared to patients with other injuries (additional file 6). In the multivariable model, having a hip fracture was associated with lower in-hospital costs (0.86 [0.78–0.94)]) but higher post-hospital costs (1.40 [1.07–1.83]) compared to other injuries (Table 4). It is important to note that in-hospital costs reflect non-surgical costs. In total, 68.4% of patients (*n* = 1307) had a surgical intervention. Of patients with hip fractures 95.6% required surgical interventions compared to 49.2% of patients without a hip fracture (additional file 6). Additionally, 72.4% of female patients had a surgical intervention compared to 65.2% of male patients (additional file 5). The need for surgical intervention fluctuated over age groups and was highest in patients ≥90 years, as 72.5% of those patients had a surgical intervention.

## Discussion

With the ageing population, the number of older trauma patients has increased. There is limited research available on the associated health care costs of this group. In this study, we described the characteristics of Dutch trauma patients older than 65 years old and their associated health care costs up to two years after injury. We found that mean costs per patient were €12,190. Older age, higher ISS, being frail and having comorbidities were associated with higher in-hospital and post-hospital costs.

Of mean total health care costs, 80% were related to leisure-related injuries, which consisted mainly of falls. The economic burden of falls in the older population is demonstrated by several other studies [12–14]. Falls are the most important cause of trauma in older people, especially women, and therefore a relevant topic for strategies for prevention. Apart from this substantial group of frail older people there are also older people, especially males, sustaining traffic or sport-related injuries. In the Netherlands, there is an increase in older road users. There is for example a growing use of electric bicycles by older adults [42]. This has resulted in an increase of the number of traffic related injuries among these older people [42, 43]. Additionally, older road users are at a higher risk of serious injury compared to younger road users, due to functional limitations and physical vulnerability [44]. A focus on the prevention of injury for older road users is therefore also advised.

Frailty was associated with in-hospital and post-hospital health care costs. After controlling for other factors including age, this association was weaker but still significant. Several other studies found a relationship between frailty and health care costs but most of these studies were cross-sectional and none were specifically focused on the older trauma population [19, 45]. Two studies in the older trauma population found no association between frailty status and hospital costs, contrary to our results [23, 24]. It is possible that the different outcome of these studies is related to the services included in the in-hospital costs, as in our study surgical costs were not included. Another possibility for the different outcomes are differences in the definition of a frail trauma patient used in the studies. Multiple frailty screening instruments and other frailty measurements exist, but only few are specific to trauma. Future research is needed to validate more existing instruments in the trauma population [46].

Women were more often admitted with a hip fracture than men and patients with hip fractures had higher post-hospital health care costs, as other studies have also shown [12]. The difference between non-surgical in-hospital costs of patients with and without a hip fracture was less profound. However, patients with hip fractures were more likely to have a surgical intervention. Mean health care costs were highest in ISS 9–15 group; however, the results of the analyses imply that these costs can partly be attributed to other factors, such as prolonged rehabilitation of injury and care needed due to their pre-existing comorbidities and/or frailty.

In the Netherlands, health insurance is mandatory, and both short-term and long-term medical care are covered by this insurance. Consistent with other studies, total health care costs were higher with older age and female sex [47, 48]. The increase in costs by age was mainly caused by post-hospital costs like homecare. Although differences in non-surgical in-hospital costs were small between men and women, we found that women had higher post-hospital costs. This can potentially be attributed to women having a higher life expectancy, more often being frail and with comorbidities and being more likely to sustain a hip fracture. Additional to the fact that we looked at the health care costs of the whole spectrum of injuries in the older trauma population in the Netherlands, two major strengths of the BIOS study were the high number of older patients who participated and the detailed information on both in-hospital and post-hospital costs and pre-injury characteristics. This study also had several limitations. Firstly, several assumptions had to be made in the calculations of costs. Mean total costs per case were calculated for all patients with available hospital or ICU costs. If other costs were missing, these were imputed with €0, which could have resulted in an underestimation of the true total costs. Additionally, in the interpretation of our results it is important to note that in-hospital costs did not include costs of surgical intervention. This has resulted in an underestimation of the in-hospital costs, specifically for patients with injuries like hip fractures, with a high percentage of surgical intervention. Secondly, the instructions of the iMCQ specifically stated to only report health care utilization related to the trauma. However, some of the reported post-hospital health care consumption may have been because of factors other than the injury. This may have led to an overestimation of the post-hospital costs. Thirdly, there is a possibility for non-response bias, since the non-responders were significantly older and more often female. This also suggests an underestimation of true costs, since both age and being female are associated with higher health care costs. Lastly, all variables had < 5% missing values except for GFI items, which were used to determine the level of frailty. The high mean health care costs of respondents with missing frailty data may indicate that older and more vulnerable patients were less likely to complete the GFI questionnaire.

## Conclusions

We conclude that the economic burden of older trauma patients is substantial. These high costs are mainly caused by high post-hospital health care consumption. This study showed that both fall-related injuries and traffic-related injuries are important areas for prevention of injuries in the older population.

## Supplementary information

**Additional file 1.** Unit costs (2017 €). Description of data: Unit costs retrieved from a Dutch cost-reference manual.

**Additional file 2.** Flow chart: Overview of BIOS study population and response on questionnaires at different times points (T). Description of data: in this flowchart, an overview of the BIOS study population is shown.

**Additional file 3.** Unadjusted associations with in-hospital, post-hospital and total health care costs based on GLM. Description of data: The unadjusted effect of patient characteristics on in-hospital, post-hospital and total health care costs.

**Additional file 4.** Mean health care costs per person by determinant for male and females in 2017 €. Description of data: Mean heath care costs by patient characteristics, separately for male and female.

**Additional file 5.** Characteristics by sex. Description of data: Characteristics of the study population, separately for male and female.

**Additional file 6.** Characteristics by injury type. Description of data: Characteristics of the study population, separately for patients with and without a hip fracture.

## Data Availability

The datasets generated and/or analysed during the current study are not publicly available because data from this study can contain potentially identifying or sensitive patient information. Data are anonymized, but due to relatively few severe cases, patients could be identified (Medical Ethics Committee Brabant). However, data are available from secretariaat@nazb.nl upon reasonable request (this is a non-author point of contact).

## Abbreviations

BIOS: Brabant Injury Outcome Surveillance
ICU: Intensive Care Unit
ED: Emergency Department
GFI: Groningen Frailty Index
BTR: Brabant Trauma Registry
AIS: Abbreviated Injury Scale
ISS: Injury Severity Score
iMCQ: iMTA Medical Consumption Questionnaire
NZa: Dutch Healthcare Authority
SD: standard deviation
GLM: generalized linear model

## References

[1] United Nations, Department of Economic and Social Affairs, Population Division (2017). World Population Ageing 2017 (ST/ESA/SER.A/408). .

[2] Prognose bevolking kerncijfers 2017–2060 [Forecast population core figures 2017–2060] [ https://opendata.cbs.nl/statline/#/CBS/nl/dataset/83783NED/table?ts=1563352557218]. Accessed 10 Nov 2019.

[3] Bevolking; generatie, geslacht, leeftijd en migratieachtergrond, 1 januari [Population;generation, sex, age and migration background, 1 January]. [https://opendata.cbs.nl/statline/#/CBS/nl/dataset/37325/table?ts=1563355478825]. Accessed 10 Nov 2019.

[4] Prognose bevolking; geslacht en leeftijd, 2019–2060 [Forecast population; sex and age, 2019–2060]. [https://opendata.cbs.nl/statline/#/CBS/nl/dataset/84346NED/table?ts=1563355080964]. Accessed 10 Nov 2019.

[5] Landelijke Netwerk Acute Zorg (LNAZ) (2018). Annual report of the dutch trauma registry: Traumazorg in beeld 2013–2017.

[6] Collard RM, Boter H, Schoevers RA, Oude Voshaar RC (2012). Prevalence of frailty in community-dwelling older persons: a systematic review. J Am Geriatr Soc.

[7] Bonne S, Schuerer DJE (2013). Trauma in the older adult: epidemiology and evolving geriatric trauma principles. Clin Geriatr Med.

[8] Keller JM, Sciadini MF, Sinclair E, O'Toole RV (2012). Geriatric trauma: demographics, injuries, and mortality. J Orthop Trauma.

[9] Geraerds A, Haagsma JA, de Munter L, Kruithof N, de Jongh M, Polinder S (2019). Medical and productivity costs after trauma. PLoS One.

[10] Weir S, Salkever DS, Rivara FP, Jurkovich GJ, Nathens AB, Mackenzie EJ (2010). One-year treatment costs of trauma care in the USA. Expert Rev Pharmacoecon Outcomes Res.

[11] Curtis K, Lam M, Mitchell R, Black D, Taylor C, Dickson C, Jan S, Palmer CS, Langcake M, Myburgh J (2014). Acute costs and predictors of higher treatment costs of trauma in New South Wales, Australia. Injury.

[12] Hartholt KA, Polinder S, Van der Cammen TJM, Panneman MJM, Van der Velde N, Van Lieshout EMM, Patka P, Van Beeck EF (2012). Costs of falls in an ageing population: a nationwide study from the Netherlands (2007–2009). Injury.

[13] Heinrich S, Rapp K, Rissmann U, Becker C, König HH (2010). Cost of falls in old age: a systematic review. Osteoporos Int.

[14] Burns ER, Stevens JA, Lee R (2016). The direct costs of fatal and non-fatal falls among older adults—United States. J Saf Res.

[15] Braithwaite RS, Col NF, Wong JB (2003). Estimating hip fracture morbidity, mortality and costs. J Am Geriatr Soc.

[16] Haentjens P, Lamraski G, Boonen S (2005). Costs and consequences of hip fracture occurrence in old age: an economic perspective. Disabil Rehabil.

[17] Leal J, Gray AM, Prieto-Alhambra D, Arden NK, Cooper C, Javaid MK, Judge A, group REs (2016). Impact of hip fracture on hospital care costs: a population-based study. Osteoporos Int.

[18] Thompson HJ, Weir S, Rivara FP, Wang J, Sullivan SD, Salkever D, MacKenzie EJ (2012). Utilization and costs of health care after geriatric traumatic brain injury. J Neurotrauma.

[19] Bock J-O, König H-H, Brenner H, Haefeli WE, Quinzler R, Matschinger H, Saum K-U, Schöttker B, Heider D (2016). Associations of frailty with health care costs – results of the ESTHER cohort study. BMC Health Serv Res.

[20] Hajek A, Bock J-O, Saum K-U, Matschinger H, Brenner H, Holleczek B, Haefeli WE, Heider D, König H-H (2017). Frailty and healthcare costs—longitudinal results of a prospective cohort study. Age Ageing.

[21] Butler A, Gallagher D, Gillespie P, Crosby L, Ryan D, Lacey L, Coen R, O'Shea E, Lawlor B (2016). Frailty: a costly phenomenon in caring for elders with cognitive impairment. Int J Geriatric Psychiatry.

[22] Lehnert T, Heider D, Leicht H, Heinrich S, Corrieri S, Luppa M, Riedel-Heller S, König H-H (2011). Health care utilization and costs of elderly persons with multiple chronic conditions. Med Care Res Rev.

[23] Kaplan SJ, Pham TN, Arbabi S, Gross JA, Damodarasamy M, Bentov I, Taitsman LA, Mitchell SH, Reed MJ (2017). Association of radiologic indicators of frailty with 1-year mortality in older trauma patients: opportunistic screening for sarcopenia and osteopenia. JAMA surgery.

[24] Min L, Ubhayakar N, Saliba D, Kelley-Quon L, Morley E, Hiatt J, Cryer H, Tillou A (2011). The vulnerable elders Survey-13 predicts hospital complications and mortality in older adults with traumatic injury: a pilot study. J Am Geriatr Soc.

[25] de Jongh MAC, Kruithof N, Gosens T, van de Ree CLP, de Munter L, Brouwers L, Polinder S, Lansink KWW (2017). Prevalence, recovery patterns and predictors of quality of life and costs after non-fatal injury: the Brabant injury outcome surveillance (BIOS) study. Injury Prev.

[26] Peters LL, Boter H, Buskens E, Slaets JPJ (2012). Measurement properties of the Groningen frailty Indicator in home-dwelling and institutionalized elderly people. J Am Med Dir Assoc.

[27] Gennarelli TA, Wodzin E (2006). AIS 2005: a contemporary injury scale. Injury.

[28] Baker SP, O'Neill B, Haddon W, Long WB (1974). The injury severity score: a method for describing patients with multiple injuries and evaluating emergency care. J Trauma Acute Care Surg.

[29] Roijen L, Linden N, Bouwmans C, Kanters T, & Tan S. (2015). Kostenhandleiding: Methodologie van kostenonderzoek en referentieprijzen voor economische evaluaties in de gezondheidszorg. Zorginstituut Nederland. The Netherlands: Zorginstituut Nederland.

[30] Passantenprijslijst 2016 [https://www6.erasmusmc.nl/cs-patientenzorg/2419534/2419543/3956735/5250821/Internetversie_passantenprijslijst_2016_v3.pdf?view=active]. Accessed 6 May 2019.

[31] BRCU -2015 Bijlage 2 Medisch specialistische behandelingen en tarieven 2011 [https://puc.overheid.nl/nza/doc/PUC_11602_22/]. Accessed 6 May 2019.

[32] Nederlandse Zorgautoriteit zorgapplicatie [https://zorgproducten.nza.nl/ZoekZorgproduct.aspx]. Accessed 6 May 2019.

[33] Standaard prijslijst overige zorgproducten [https://www.elkerliek.nl/elkerliek.nl/home/Passantanprijslijst%20Overige%20Zorgproducten%2001042013%20tm%2030062013.pdf]. Accessed 6 May 2019.

[34] Tarievenlijst Eerstelijnsdiagnostiek [https://docplayer.nl/13384312-Tarievenlijst-eerstelijnsdiagnostiek-bijlage-1-bij-tariefbeschikking-tb-cu-7078-02-van-1-juni-2014.html]. Accessed 6 May 2019.

[35] Bio-impedantiemeting [https://www.rivas.nl/voeding-leefstijl/gezondheidsmetingen/cursus/bio-impedantiemeting/?tx_roqcursussenactiviteiten_cursussen%5Baction%5D=show&tx_roqcursussenactiviteiten_cursussen%5Bcontroller%5D=Course&cHash=094076003728918c33f5113ca52651f1]. Accessed 6 May 2019.

[36] Passanten prijslijst DBC-OVP zorgproducten [https://zeelandcare.com/sites/default/files/Prijslijst%20Passanten%20DBC-OVP%20zorgproducten%202018.pdf]. Accessed 6 May 2019.

[37] Upatising B, Wood DL, Kremers WK, Christ SL, Yih Y, Hanson GJ, Takahashi PY (2015). Cost comparison between home telemonitoring and usual care of older adults: a randomized trial (Tele-ERA). Telemed J E Health.

[38] Barber J, Thompson S (2004). Multiple regression of cost data: use of generalised linear models. J Health Serv Res Policy.

[39] Sterne JAC, White IR, Carlin JB, Spratt M, Royston P, Kenward MG, Wood AM, Carpenter JR (2009). Multiple imputation for missing data in epidemiological and clinical research: potential and pitfalls. Bmj.

[40] Eekhout I, de Vet HCW, Twisk JWR, Brand JPL, de Boer MR, Heymans MW (2014). Missing data in a multi-item instrument were best handled by multiple imputation at the item score level. J Clin Epidemiol.

[41] Buuren S, Groothuis-Oudshoorn K. MICE: Multivariate Imputation by Chained Equations in R. J Stat Softw. 2011;45(3):1–67. .

[42] Poos H, Lefarth TL, Harbers JS, Wendt KW, El MM, Reininga IHF (2017). E-bikers are more often seriously injured in bicycle accidents: results from the Groningen bicycle accident database. Ned Tijdschr Geneeskd.

[43] Weijermars W, Bos N, Stipdonk HL (2016). Serious road injuries in the Netherlands dissected. Traffic Injury Prev.

[44] European Commission (2015). Older Drivers. Directorate General for Transport.

[45] Peters LL, Burgerhof JGM, Boter H, Wild B, Buskens E, Slaets JPJ (2015). Predictive validity of a frailty measure (GFI) and a case complexity measure (IM-E-SA) on healthcare costs in an elderly population. J Psychosom Res.

[46] Maxwell CA, Patel MB, Suarez-Rodriguez LC, Miller RS (2019). Frailty and prognostication in geriatric surgery and trauma. Clin Geriatr Med.

[47] Polinder S, Haagsma J, Panneman M, Scholten A, Brugmans M, Van Beeck E (2016). The economic burden of injury: health care and productivity costs of injuries in the Netherlands. Accid Anal Prev.

[48] Corso P, Finkelstein E, Miller T, Fiebelkorn I, Zaloshnja E (2006). Incidence and lifetime costs of injuries in the United States. Inj Prev.

