# Supplementary material for: Health care costs of injury in the older population: a prospective multicentre cohort study in the Netherlands
Source: BMC Geriatr. 2020 Oct 21;20:417. doi: 10.1186/s12877-020-01825-z (PMC7576762; doi:10.1186/s12877-020-01825-z)
Supplement: Supplementary file 2 — Additional file 2. Flow chart: Overview of BIOS study population and response on questionnaires at different times points (T). Description of data: in this flowchart, an overview of the BIOS study population is shown. [file 12877_2020_1825_MOESM2_ESM.docx]

Eligible ≥65 years patients BIOS

(N=5504)

Total study population

(N=1910, 34.7%)

Exclusion:

No response (N=3437)

No data on in-hospital care (N=157)

iMCQ 1 month after injury

(N= 1426, 74.7%)

iMCQ 3 month after injury

(N=1430, 74.9%)

iMCQ 6 month after injury

(N=1272, 66.6%)

iMCQ 12 month after injury

(N=1223, 64.0%)

Died between T1 and T2

(N=1, 0.1%)

Died between T2 and T3

(N=51, 2.7%)

Died between T3 and T4

(N=49, 2.6%)

Died between T4 and T5

(N=68, 3.6%)

Died between T5 and T6

(N=65, 3.4%)

iMCQ 24 month after injury

(N=1067, 55.9%)

**Figure A.1. Flow chart: Overview of BIOS study population and response on questionnaires at different times points (T).**
